# Morphology and Ciliary Motion of Mucosa in the Eustachian Tube of Neonatal and Adult Gerbils

**DOI:** 10.1371/journal.pone.0099840

**Published:** 2014-06-12

**Authors:** Yi Li, Huizhan Liu, Jun Li, Qian Zhang, Shusheng Gong, David He

**Affiliations:** 1 Department of Otolaryngology―Head and Neck Surgery, Beijing Tongren Hospital, Capital Medical University, Beijing, P.R. China; 2 Department of Biomedical Sciences, Creighton University School of Medicine, Omaha, Nebraska, United States of America; 3 Department of Otolaryngology, Hospital of Zhongshan Qingpu, Fudan University, Shanghai, P.R. China; University of South Florida, United States of America

## Abstract

The Eustachian tube is a small canal that connects the tympanic cavity with the nasal part of the pharynx. The epithelial lining of the Eustachian tube contains a ciliated columnar epithelium at the tympanic cavity and a pseudostratified, ciliated columnar epithelium with goblet cells near the pharynx. The tube serves to equalize air pressure across the eardrum and drains mucus away from the middle ear into the nasopharynx. Blockage of the Eustachian tube is the most common cause of all forms of otitis media, which is common in children. In the present study, we examined the epithelial lining of the Eustachian tube in neonatal and adult gerbils, with a focus on the morphological and functional development of ciliated cells in the mucosa. The length of the tube is ∼8.8 mm in adult gerbils. Scanning electron microscopy showed that the mucosal member near the pharyngeal side contains a higher density of ciliated cells and goblet cells than that near the tympanic side. The cilia beat frequency is 11 Hz. During development, the length of the Eustachian tube increased significantly between postnatal day 1 (P1) and P18. Scanning electron microscopy showed that the mucosa contained a high density of ciliated cells with a few goblet cells at P1. The density of ciliated cells decreased while the density of goblet cells increased during development. At P18, the mucosa appeared to be adult-like. Interestingly, the ciliary beat frequency measured from ciliated cells at P1 was not statistically different from that measured from adult animals. Our study suggests that the Eustachian tube undergoes significant anatomical and histological changes between P1 and P18. The tube is morphologically and functionally mature at P18, when the auditory function (sensitivity and frequency selectivity) is mature in this species.

## Introduction

The Eustachian tube (ET) is a small canal that connects the tympanic cavity with the nasal part of the pharynx. The ET acts as a passageway to ventilate the tympanic cavity and allows equalization of pressure between the middle ear and pharynx, which is necessary for normal hearing [1]. Additionally, it drains mucus away from the middle ear into the nasopharynx. Abnormal or impaired function(s) of the ET may cause pathological changes in the middle ear. Blockage of the ET is the most common cause of all forms of otitis media [2], which is common in children, partly because the ET is not completely developed.

Although the development of some parts of the murine middle ear has been described [3], [4], to date, knowledge of the morphological and functional development of the ET mucociliary system is sketchy. In this study, our goal was to examine the epithelial lining of the ET in neonatal and adult gerbils, with a focus on the morphological and functional development of ciliated cells in the mucosa. We examined anatomical and ultrastructural changes of the ET and its epithelial lining, the mucosa, in neonatal and adult gerbils. Since ciliary motion of the mucosa is important for drainage of the ET and impaired mucociliary function of the ET and respiratory tract mucosa is associated with secretory otitis media [5], we also measured ciliary beat frequency of the ciliated cells at different locations along the ET in neonatal and adult gerbils using a photodiode-based displacement measurement system. Our study not only showed how the ET and its mucosa develop, but also provides a morphological and functional basis for future animal studies concerning the pathogenesis of otitis media in earlier stages using the murine model.

## Materials and Methods

### Animals

Neonatal gerbils aged between postnatal day 1 (P1) and P30 were used for the experiments. The animals were euthanized by an overdose of sodium pentobarbital (200 mg/kg, IP) followed by decapitation. The animals were used according to a protocol approved by the Institutional Animal Care and Use Committee of Creighton University.

### Histological Examinations of the ET

Temporal bone was isolated at the ages of P1, P8, P12, P18, and P30. The tissue was fixed in 4% paraformaldehyde in PBS at 4°C overnight. After being washed with PBS, the tissue was embedded in celloidin. Serial cross sections (10 µm) were prepared with a microtome (CM3050, Leica, Nussloch, Germany) and collected on microslides. The sections were stained with hematoxylin and eosin. After washing with PBS solution, the slides were mounted with a glycerol-based mounting solution and examined under a microscope. The length of the ET was determined using ImageJ (version 1.47) from captured images.

### Scanning Electron Microscopy

For scanning electron microscopy (SEM), the temporal bone tissues were fixed with 2.5% glutaraldehyde in 0.1 M sodium cacodylate buffer (pH 7.4) containing 2 mM CaCl_2_, washed in PBS, then post-fixed for 15 minutes with 1% OsO_4_ in the same buffer and washed. The tissues were dehydrated in an ethanol series, critical point-dried from CO_2_, and sputter-coated with gold. The tissue was then examined using an FEI Quanta200 scanning electron microscope.

### Measurements of Ciliary Motion

The ET was dissected out and sectioned along its longitudinal length. The preparation was bathed in L-15 medium (Invitrogen), containing 136 mM NaCl, 5.8 mM NaH_2_PO_4_, 5.4 mM KCl, 1.4 mM CaCl_2_, 0.9 mM MgCl_2_, 0.4 mM MgSO_4_, and 10 mM HEPES-NaOH (pH 7.4, 300 mmol/l) in an experimental chamber mounted on the stage of a Leica upright microscope. The tissue was firmly attached to the bottom of the chamber by the weight of two thin platinum rods (0.5 mm in diameter), with one of their ends anchored in two small droplets of vacuum grease on the bottom of the chamber. The tissue was mounted with the hair bundle facing upward toward the water-immersion objective. The cilia were imaged using a 63× water immersion objective (Leica) and magnified by an additional 20× relay lens. Ciliary motion was measured and calibrated by a photodiode-based measurement system [6], [7] mounted on the Leica upright microscope. The magnified image of the cilia was projected onto a photodiode through a rectangular slit. Spontaneous cilia motion modulated the light influx to the photodiode. The photocurrent response was calibrated to displacement units by moving the slit a fixed distance (0.5 µm) with the image of the cell in front of the photodiode. After amplification, the photocurrent signal was low pass-filtered by an antialiasing filter before being digitized by a 16-bit A/D board (Digidata 1322; Molecular Devices). The photodiode system had a cutoff (3 dB) frequency of 1,100 Hz. The motile responses were filtered at 200 Hz and digitized at 10 kHz. Spontaneous ciliary motion was acquired in a four-second window and five of such response segments were obtained in each run. The power spectrum of the response was averaged and analyzed in the frequency domain using Clampfit software. The experiments were done at room temperature (22±2°C).

## Results

### 1. Morphology of the ET and Its Mucosal Epithelium in Adult Gerbils

**Figure 1:**
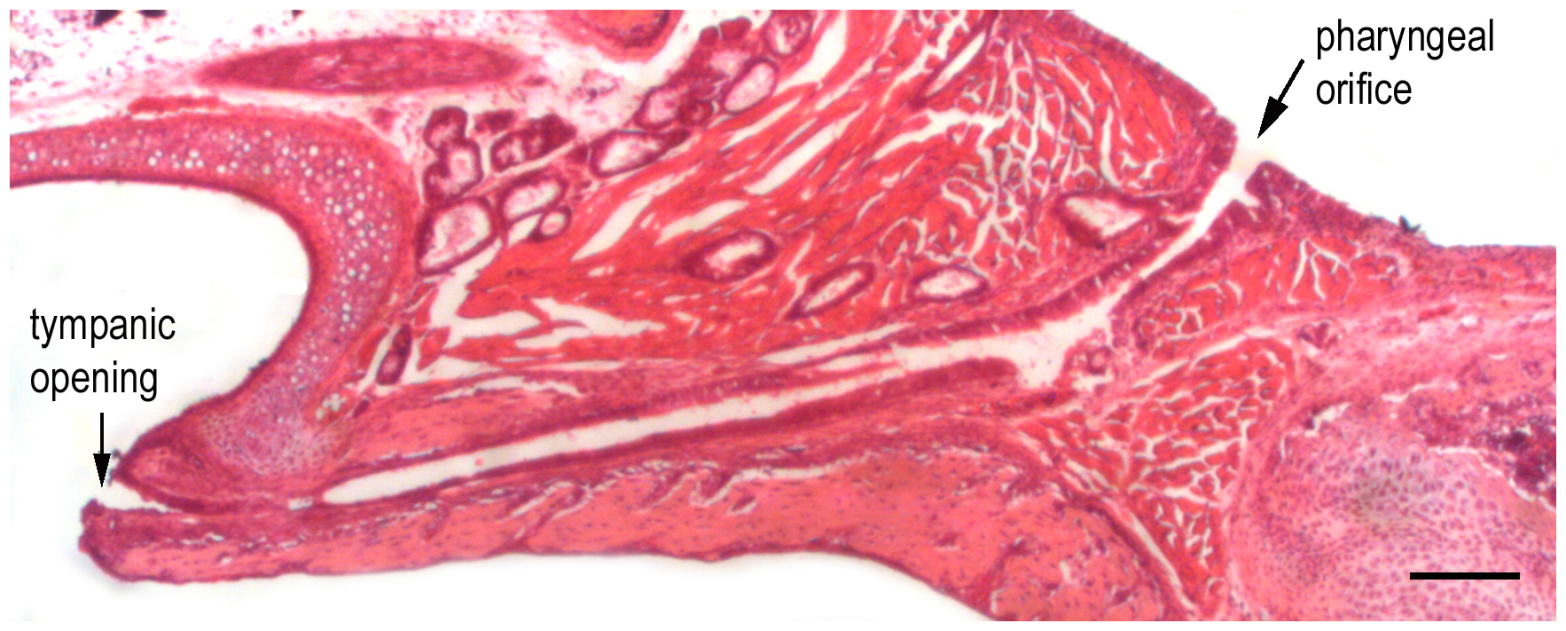
Longitudinal section of the TB of a 30-day-old gerbil stained with hematoxylin and eosin. The length of the Eustachian tube measured between the tympanic and pharyngeal ends in adult gerbil is ∼0.8 mm. Bar: 50 µm.

The auditory system is mature after P20 in gerbils. We measured the length of the ET in the cross-section preparation (Fig. 1) of the adult gerbil at P30. The length of the ET, measured between the tympanic and pharyngeal ends (marked by arrows in Fig. 1), was 0.88±0.9 mm (n = 7). The ET is often divided into two portions: the osseous portion and the cartilage portion. The osseous portion constitutes approximately one-third of the entire length of the ET. We examined the histological architecture of the mucosal epithelium of the ET in the osseous portion near the tympanic opening and the cartilage portion near the pharyngeal opening from three animals using light microscopy. The mucosal epithelium near the pharyngeal opening is lined with pseudostratified ciliated columnar epithelium composed of ciliated and goblet cells. There is a gradual change to a mixture of ciliated, goblet, and squamous cells near the tympanic opening. The density of ciliated cells and goblet cells near the pharyngeal opening appears to be higher than that in the area near the tympanic opening.

**Figure 2:**
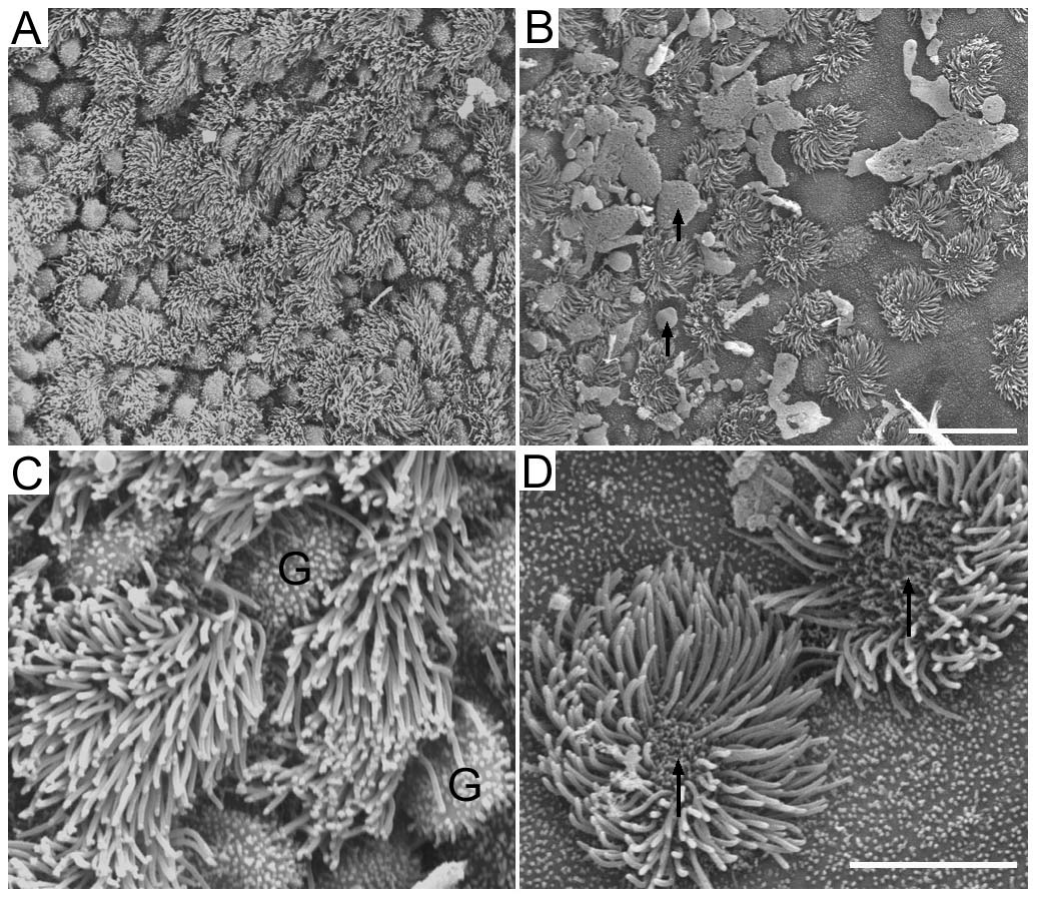
SEM pictures of ciliated epithelium near the pharyngeal opening (A) and tympanic opening (B) of the ET from a P30 gerbil. Note that the density of ciliated cells near the pharyngeal opening is significantly greater than that near the tympanic opening. Black arrows indicate mucus drops. Bar: 15 µm for panels A and B. C: High magnification picture of ciliated cells and goblet cells in the area near the pharyngeal opening. G stands for goblet cells. D: High magnification picture of ciliated cells and squamous cells in the area near the tympanic opening. Note that there was absorption of cilia (marked by black arrows). Bar: 5 µm for C and D.

We examined the ultrastructural change of the mucosal epithelium in two locations approximately 60 µm near the tympanic and pharyngeal openings from three animals using SEM. As illustrated in Fig. 2, the mucosal epithelium contains ciliated cells and goblet cells in both regions. However, the density of ciliated cells and goblet cells in the different regions are different. Consistent with the light microscopic observation, the mucosa in the region near the pharyngeal opening contains a higher density of ciliated cells and goblet cells. The region near the tympanic opening has fewer ciliated cells. We counted the total number of ciliated cells in a 150×75 µm area in both regions from three ET preparations from three gerbils. The number of ciliated cells is 126±16 and 61±10 for the pharyngeal and tympanic regions, respectively. Thus the density of ciliated cell is significantly higher in the pharyngeal region than in the tympanic region (p<0.01, student's t-test). More squamous cells were observed in this area. Squamous cells have short blunt microvilia. We also observed absorption of cilia in ciliated cells in both regions. In all three samples examined, the absorption of cilia all started from the center of the cilia (marked by arrows in Fig. 2D). However, we did not observe any signs of the growth of new cilia. The fate and identity of the ciliated cells after the cilia were absorbed are unclear.

### 2. Ciliary Beat Frequency of Ciliated Cells

**Figure 3:**
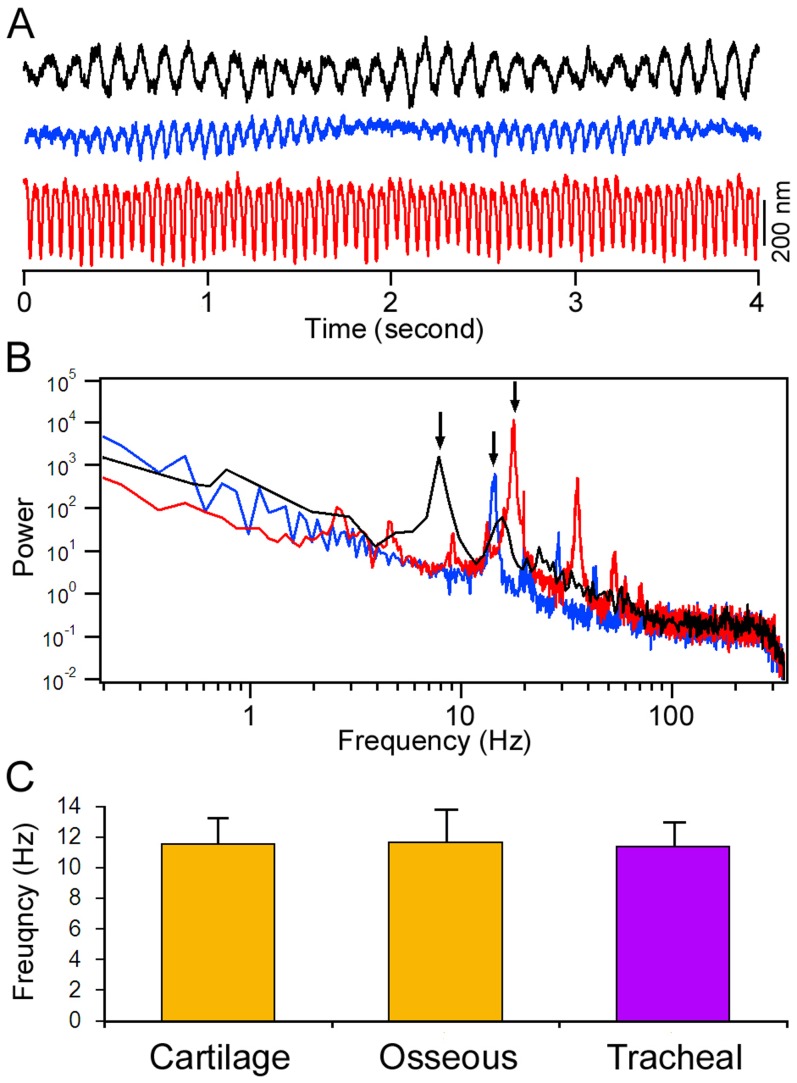
Ciliary motion measured from adult gerbils. A: Three representative waveforms of spontaneous ciliary motion measured from three different ciliated cells near the pharyngeal opening using a photodiode-based displacement measurement system. Different patterns of responses with different frequencies and magnitudes were observed. B: The power spectra of cilia motion. The spectra represent five averages (in the frequency domain) of spontaneous ciliary response acquired in a four-second window in each trial. C: Means and SDs of ciliary beat frequency. The responses were measured from 15 ciliated cells in the cartilage and osseous portion of the ET from five animals. For comparison, ciliary motion was also measured from 12 ciliated cells in the airway of three adult gerbils. No statistical difference (p>0.05) of the beat frequency was found among ciliated cells in the different regions.

Ciliary motion of the mucosal epithelium in the ET is important for clearance. We measured and compared ciliary beat frequency from ciliated cells located in the osseous and cartilage portions of the ET from five adult gerbils (P30). Fig. 3A shows three representative response waveforms of ciliary motions from three different ciliated cells near the pharyngeal orifice in the cartilage portion. As shown, different cells exhibit ciliary motions with different frequencies, magnitudes, and patterns. We analyzed the beat frequency using a fast Fourier transform. The frequency spectrum of the responses is presented in Fig. 3B. All three responses have a main frequency component between 8 to 15 Hz, with several harmonics at higher frequencies in the spectrum. Fig. 3C presents the means and standard deviations of the frequency of the main frequency component measured from 15 cells in the cartilage portion. For comparison, we also measured the frequency of the main frequency component from 15 ciliated cells in the osseous portion and 12 cells in the airway tissue [7]. As shown in Fig. 3C, the beat frequency of the ciliated cells in the osseous and cartilage portions is not statistically different (p>0.05). No statistical difference (p>0.05) in beat frequency was found between the ciliated cells in the ET and airway tissue, either.

### 3. Development of the ET and Its Mucosal Epithelium

**Figure 4:**
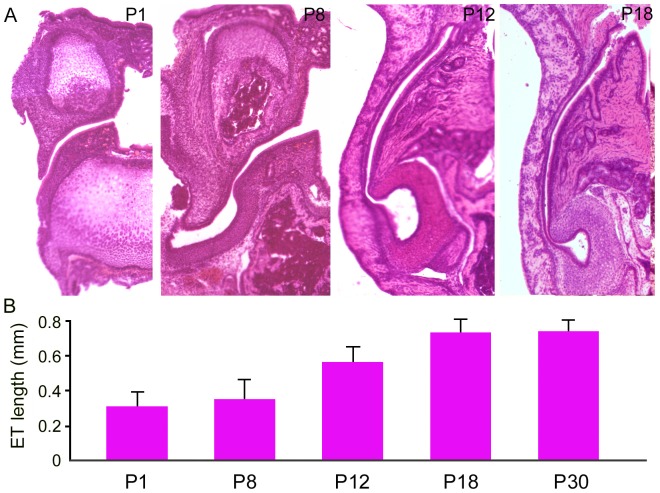
Morphological development of the ET. A: Longitudinal sections prepared from neonatal gerbils at P1, P8, P12, P18, and P30. B: Means and SDs of length of the ET (measured between the pharyngeal opening and the tympanic opening). The measurements were made from five temporal bone preparations from five animals for each age group.

Gerbils, like mice and rats, are altricial animals and their auditory system develops within the first three weeks after birth. We examined anatomical and morphological changes of the ET between P1 and P30 from five animals for each age group. Fig. 4 shows the development of length of the ET between P1 and P30. As shown, the length of the ET grows significantly during this time window. The significant growth occurs after P8; by P18, the length of the ET is not statistically different from that of the adult animals (P>0.05).

**Figure 5:**
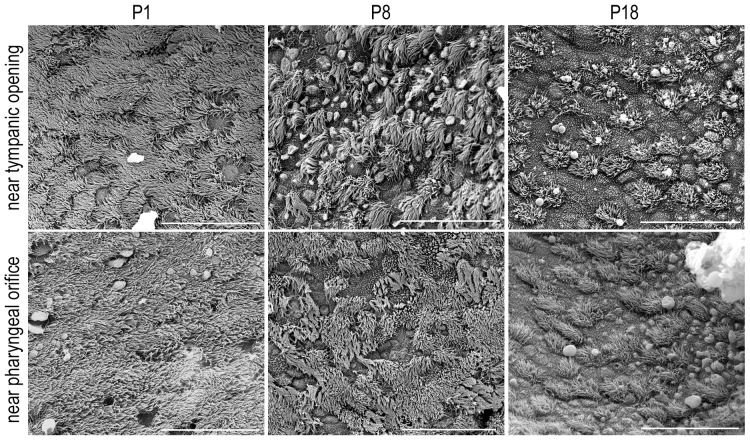
SEM pictures of the mucosal epithelium acquired from two areas near the pharyngeal and tympanic openings in the ET during development. Bars: 30 µm.

We also examined the ultrastructural changes of the mucosal epithelium in the osseous and cartilage regions of the tube at P1, P8, P18, and P30 using SEM. Three animals for each age group were examined. As illustrated in Fig. 5, the mucosal epithelium undergoes significant change during this time window. At P1, both regions were covered by densely populated ciliated cells; only a few goblet cells were seen. The density of the ciliated cells decreased while the density of goblet cells increased during development in the cartilage region near the pharyngeal orifice. In the osseous portion, there was an increase in the density of squamous cells and a reduction in the density of the ciliated and goblet cells. We were unable to count the ciliated cells at P1 due to the fact that the mucosal epithelium was almost completely covered by cilia. However, we counted the total number of ciliated cells in the two regions at P8 and P18 from three ET preparations. In both regions, there was a significant reduction of the density of ciliated cell between P8 and P18 (p<0.05). By P18, the mucosal membrane reached adult appearance.

### 4. Ciliary Beat Frequency of Ciliated Cells in Neonatal Gerbils

**Figure 6:**
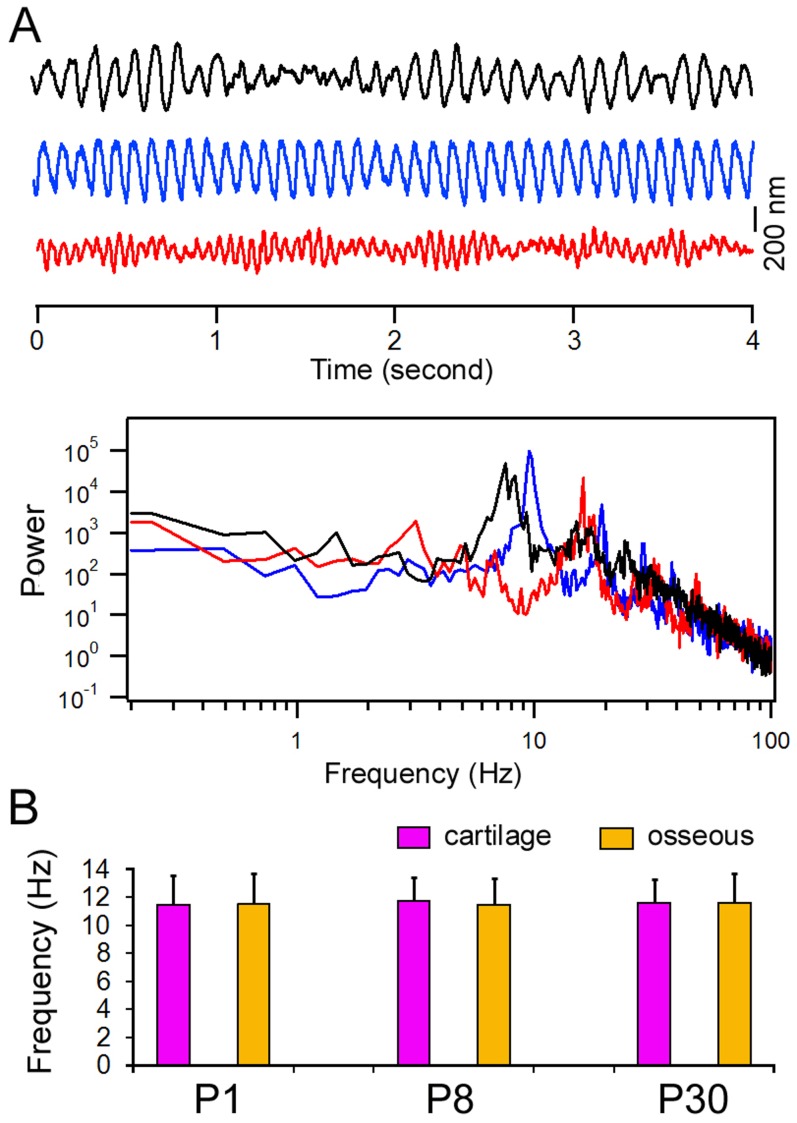
Spontaneous ciliary activity during development. A: Three representative waveforms of spontaneous ciliary motion measured from ciliated cells near the pharyngeal opening using a photodiode-based displacement measurement system. The responses were obtained from three different cells at P1. B: The power spectra of ciliary motion of the waveforms shown in Panel A. C: Means and SD of beat frequencies during different stages of development. The means are based on measurements made from 12 cells from three animals for each age group.

Although ciliary beat frequency has been measured in adult animals, the development of ciliary motion has not been examined. We measured ciliary motion from ciliated cells in the cartilage region at different time points during development. Fig. 6A shows some representative response waveforms from three different cells at P1. Similar to the responses seen in adult animals, the ciliary motion in neonatal gerbils also exhibited different patterns, frequencies, and magnitudes. The frequency spectrum of the response is presented in Fig. 6B. Fig. 6C illustrates the mean and standard deviations of the beat frequency (main frequency component) measured from 12 cells in the cartilage portion at P1, P8, P18, and P30. As shown, the beat frequency at P1 is not statistically different from that of P8, P18, and P30. In other words, the ciliary motion is developed at birth.

## Discussion

We used morphological and electrophysiological methodology to examine the mucosal epithelium in neonatal and adult gerbils. Gerbil is one of the most commonly used animal models for auditory research. Like other rodents, the middle ear and inner ear of gerbils develop after birth [8]. Previous studies using developing gerbils showed that cochlear microphonic responses were first recorded at P12, with thresholds exceeding 100 dB SPL, suggesting that the onset of hearing occurs around P12 [9]. Cochlear microphonic thresholds subsequently improved rapidly across the responsive frequency range, achieving adult levels by P18 [9]. Although other studies suggest that middle ear and inner ear functions (such as cochlear potentials and basilar membrane mechanics) continue to improve beyond P30 [10], [11], it is generally believed that the gerbil auditory system is functionally and morphologically mature at P18. Although we did not examine gross morphology of the middle ear, our study shows that the development of the ET, which is considered part of the middle ear, occurs before P18. The most dramatic increase in ET length is observed between P8 and P18. This period is associated with the final stages of resorption of middle ear mesenchyme and ossicular ossification, and accounts for approximately a 25 dB loss in sensitivity due to middle ear immaturity [12]. As one of the major roles of the ET is to ventilate the tympanic cavity to allow equalization of pressure between the middle ear and pharynx, concurrent development of the ET is necessary for functional maturation of the auditory system in this species.

Using SEM, we show that there is a gradual change of the mucosal membrane along the ET in both neonatal and adult animals. The structure of the gerbil mucosa shows striking similarities to that of mice, rats, guinea pigs, dogs, and humans [13]–[16]. In the cartilage region near the pharyngeal opening, there is a higher density of ciliated cells and goblet cells. The density of ciliated cells is reduced significantly in the tympanic region. Such gradual change of the ciliated cell density in mucosal epithelium has been reported before. Interestingly, we observed a dynamic change of the cilia in both regions; such change is reflected by resorption of cilia (Fig. 2). It is not clear, however, whether such resorption of cilia is a sign of the beginning of the renewal of the cilia or the cell. Mature cells in the epithelium have a different life span; the turnover time of ciliated cells and goblet cells in guinea pigs is estimated to be six days [17].

Ultrastructural examination shows that the mucosal epithelium of the ET contains a higher density of ciliated cells at birth. Studies in rats and mice have shown that development of ciliated cells starts at the sixteenth gestation day [3], [18]–[20], one day earlier than the secretory cells appear in the ET and middle ear. The number of ciliated cells and secretory cells increases rapidly after birth. Although we did not examine the development of the mucosal epithelium before birth, we observed a higher density of ciliated cells in the ET in neonatal animals than in the adult animals; the high density of ciliated cells remained until P8. However, contrary to the study by Park and Lim in mice [20], we observed a significant reduction of ciliated cells between P8 and P18. In the area near the tympanic opening, the reduction was even more dramatic. The significant remodeling of the mucosal epithelium after P8 is more consistent with the ultrastructure of mucosa observed in adult animals.

Fluid and debris are continually being cleared and drained from the middle ear through the ET by mucociliary clearance [21], [22]. The mucociliary transport system is a major defense mechanism of the middle ear. Direct measurement of mucociliary clearance in the ET of small animals such as gerbils has never been done. To measure this clearance in neonatal gerbils is more difficult since the middle ear is filled with mesenchyme before P10. Thus, the performance of mucous transportation in the middle ear and ET is often represented by measuring ciliary motion. Cilia beat frequency has been measured in animals [23] and humans [5], [24], [25] under normal and pathological conditions. The beat frequency ranges from 8 to 15 Hz. We demonstrated that the mean beat frequency of the ciliated cells in the ET was 11 Hz, consistent with that seen in biopsy tissue obtained from the middle ear of children [24]. Ciliary motion is known to be sensitive to temperature, pH, calcium, and ATP concentration [26]–[28]. Drugs (e.g., cocaine, adrenaline), smoking (nicotine), infections (viral or bacterial), and noxious fumes (e.g., sulfur, carbon monoxide) all inhibit the normal ciliary beat [28]. Under our experimental condition, the beat frequency of cilia in the different regions of the ET was similar, despite the fact that there was gradual change in cilia density along the length of the ET. We further showed that cilia beat in the ET was not significantly different from that seen in the airway [7]. The methods that have been used most frequently to measure ciliary beat frequency in the middle ear and airway tissues are the cinematograph, high-speed digital camera [29], [30], and more recently, the optical flow technique [31] and optical coherence tomography [32]. We used the photodiode technique to measure cilia motion. This technique has some advantages over other techniques. While other techniques can quantitatively measure beat frequency, complex response waveforms with harmonic components are often missed. We showed, with power spectrum analysis, that the cilia motion was complex, with different frequencies, magnitudes, and patterns. The disadvantage of the photodiode technique is that the measurement can only be made from one cell at a time, whereas the optical flow technique and optical coherence tomography can capture motions of all cells in a larger area. Optical coherence tomography has recently been used to measure functional dynamics of cilia and mucus flow on the airway epithelium [32].

Cilia beat frequency of the ciliated cells from the middle ear has not been measured from developing animals. Therefore, it is unclear whether cilia motion is mature at birth or still undergoes development in the first three weeks after birth. We measured cilia motion at different time points at two different areas in the ET during development and showed that cilia beat frequency measured at birth was not statistically different from that measured from adult animals. The magnitude and pattern of motion are compatible among different age groups. Combined with the ultrastructural evidence using SEM, we conclude that ciliated cells are morphologically and functionally mature at birth.

In summary, we examined the morphology and function of the mucosa in the ET of neonatal and adult gerbils in the present study. We show that the ET undergoes significant anatomical and histological changes between P1 and P18. The tube is morphologically and functionally mature at P18, when the auditory function is mature in this species. Although the anatomy (in terms of length) and development of the ET in humans and gerbils are different, our study provides important information about ultrastructural and functional changes of the mucosa in the ET of neonatal and adult gerbils, which may help to understand the development and function of the ET in humans.
